# Association between Early Life Child Development and Family Dog Ownership: A Prospective Birth Cohort Study of the Japan Environment and Children’s Study

**DOI:** 10.3390/ijerph18137082

**Published:** 2021-07-02

**Authors:** Machiko Minatoya, Atsuko Ikeda-Araki, Chihiro Miyashita, Sachiko Itoh, Sumitaka Kobayashi, Keiko Yamazaki, Yu Ait Bamai, Yasuaki Saijo, Yukihiro Sato, Yoshiya Ito, Reiko Kishi, The Japan Environment and Children’s Study Group

**Affiliations:** 1 Center for Environmental and Health Sciences, Hokkaido University, Sapporo 060-0812, Japan; mminatoya@cehs.hokudai.ac.jp (M.M.); aaraki@cehs.hokudai.ac.jp (A.I.-A.); miyasita@med.hokudai.ac.jp (C.M.); vzbghjn@den.hokudai.ac.jp (S.I.); sukobayashi@cehs.hokudai.ac.jp (S.K.); kyamazaki@cehs.hokudai.ac.jp (K.Y.); u-aitbamai@med.hokudai.ac.jp (Y.A.B.); 2 Faculty of Health Sciences, Hokkaido University, Sapporo 060-0812, Japan; 3 Department of Social Medicine, Asahikawa Medical University, Asahikawa 078-8510, Japan; y-saijo@asahikawa-med.ac.jp (Y.S.); yukihiro-sato@asahikawa-med.ac.jp (Y.S.); 4 Faculty of Nursing, Japanese Red Cross Hokkaido College of Nursing, Kitami 090-0011, Japan; yito@rchokkaido-cn.ac.jp

**Keywords:** family dog ownership, child development, birth cohort

## Abstract

Pets may play a role in the social-emotional development of children. In particular, some studies have suggested that family dog ownership is associated with better health outcomes. To date, no study has assessed child development in association with dog ownership of different time points. The purpose of the current study was primary to investigate whether “ever” family dog ownership was associated with early child development, and secondary to further examine whether associations between family dog ownership and early child development differ among family dog ownership of status, including “past only”, “current only”, and “always” groups, using the data of family dog ownership obtained at multiple time points. Associations between family dog ownership and infant development at 3 years of age were examined using data from a nationwide prospective birth cohort study, the Japan Environment and Children’s Study (*n* = 78,941). “Ever” family dog ownership was categorized to “past only”, “current only”, and “always”. We observed that children with “ever” family dog ownership showed a significantly decreased risk of developmental delay in the communication (odds ratio [OR] = 0.87; 95% confidence interval [CI]: 0.78, 0.96), gross motor (OR = 0.83; 95% CI: 0.76, 0.92), problem-solving (OR = 0.89; 95% CI: 0.83, 0.96) and personal-social (OR = 0.86; 95% CI: 0.72, 0.92) domains compared to children with “never” family dog ownership. Furthermore, a significantly decreased risk of developmental delay in gross motor function was observed in association with living with dogs in the “past only” (OR = 0.83; 95% CI: 0.73, 0.95) and “always” (OR = 0.86; 95% CI: 0.75, 0.98). In addition, a decreased risk of developmental delay in the problem-solving domain was associated with “past” family dog ownership (OR = 0.87; 95% CI: 0.79, 0.97) and in the personal-social domain was associated with “always” family dog ownership (OR = 0.81; 95% CI = 0.68, 0.95). Given the possible positive association between early life child development and family dog ownership, living with dogs may be an important factor to be considered when assessing child development.

## 1. Introduction

Prior research has demonstrated that pet ownership is associated with physical, psychological, and social benefits among children [1,2]. In particular, pets may play a role in the social-emotional development of children, such as in the development of self-esteem, autonomy, and empathy for others [3]. Attachment to a pet may impact emotional development [4]. A positive relationship between emotional bonds with pets and youth social-emotional outcomes has been reported [5]. Further, a number of studies have suggested that family dog ownership is associated with better health outcomes. For instance, family dog ownership is associated with walking and physical activity in school children [6]. However, these studies had the limitation of a cross-sectional design that precluded assessment of the causal relationship between family dog ownership and beneficial health outcomes. In addition, several studies have found associations between pet keeping and neurodevelopmental disorders or measures of related symptoms; however, their findings may have been confounded by exposure misclassification [7] and confounding factors [8]. In addition to the family dog ownership settings, several randomized controlled studies have shown the positive effects of animal therapy. A clinical trial showed that increased social support through pet ownership lowered blood pressure response to mental stress [9], and a randomized controlled trial study found that the use of an animal-assisted therapy (AAT) program increased the well-being of university students experiencing homesickness [10]. An animal-assisted reading program had an impact on the reading skills of students who read to a dog [11]. Similar to the aforementioned studies, cross-sectional and randomized controlled studies have reported positive associations; however, limited longitudinal studies have investigated associations between family dog ownership and child developmental outcomes. 

In our recent study using data from a prospective birth cohort study, having a dog at 6 months of age was associated with decreased risks of infant developmental delays at 12 months of age, which was observed only among dog owners but not cat owners [12]. This previous study investigated family dog ownership only at one point in time; however, examination of the duration of family dog ownership and the critical time period for child development would be desirable for a better understanding of the previous findings. To the date, no study has assessed child development in association with dog ownership of different time points. These limitations highlighted the importance of investigating child development in association with family dog ownership of different and multiple time points. The purpose of the current study was primarily to investigate whether “ever” family dog ownership was associated with early child development, and second to further examine whether associations between family dog ownership and early child development differ among family dog ownership of status, including “past only”, “current only”, and “always” groups using the data of family dog ownership obtained at multiple time points from a nationwide prospective birth cohort study, the Japan Environment and Children’s Study (JECS).

## 2. Materials and Methods

### 2.1. Study Design

The JECS is an ongoing, nationwide prospective birth cohort study. The study was conducted at 15 regional centers (Hokkaido, Miyagi, Fukushima, Chiba, Kanagawa, Koshin, Toyama, Aichi, Kyoto, Osaka, Hyogo, Tottori, Kochi, Fukuoka, and Minami Kyusyu/Okinawa) in Japan. The details of the JECS project have been described elsewhere [13,14,15]. Briefly, pregnant women were recruited between January 2011 and March 2014. The eligibility criteria for participation included residing in the study area at the time of recruitment, an expected delivery date after August 2011, comprehension of the Japanese language, and completion of the self-administered questionnaire. In total, 104,062 registered children were included in the cohort, including multiple births. The present study used the dataset jecs-ta-201901930-qsn, which was released in October 2019 and revised in February 2020.

### 2.2. Ethical Statement

The JECS protocol was approved by the Ministry of the Environment’s Institutional Review Board on Epidemiological Studies and by the ethics committees of each participating institution (Appendix A) (ethical project identification code: Kanken19–117). All participants provided informed written consent in accordance with the Declaration of Helsinki (sixth version).

### 2.3. Study Population

**Figure 1:**
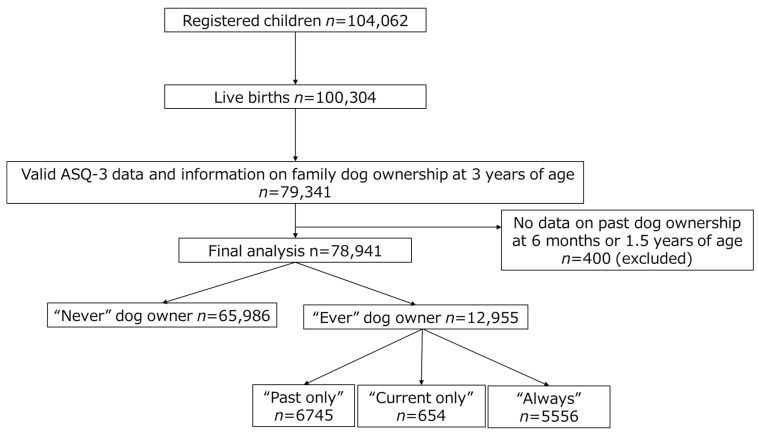
Selection of the study population.

Of the 104,062 pregnant women included in the cohort, 100,304 had live births. Among the JECS participants, those with available data for Ages and Stage third edition (ASQ-3), family dog ownership at 3 years of age, and information regarding family dog ownership at 6 months and/or 1.5 years of age were included in this study (*n* = 78,941) (Figure 1).

### 2.4. Self-Administered Questionnaires

Details of the self-administered questionnaire used in this study have been described previously [13,14]. Briefly, data on maternal smoking and drinking at the second/third trimester, maternal and paternal education, and annual family income during pregnancy were obtained from the M-T2 questionnaire (answered by pregnant women at the second/third trimester); data on parity were obtained from the Dr-T1 questionnaire (medical records transcripts at the first trimester); and data on maternal age at delivery, delivery mode, infant sex, gestational age, and birth weight and length were obtained from the Dr-0m questionnaire (medical records transcripts at delivery). Data on maternal health-related quality of life (HRQOL) assessed using Short Form-8 (SF-8) were obtained from the C2.5Y questionnaire (answered by mothers at 2.5 years postpartum). Data on the weight and height of children, information on daycare attendance, family income, maternal and paternal smoking status, and environmental tobacco smoke exposure (ETS) were obtained from the C-3Y questionnaire (answered by mothers at 3 years postpartum).

### 2.5. Exposure Definitions

Information regarding past family ownership of the dog was obtained from the C-6M and C-1.5Y questionnaires, and current information was obtained from the C-3Y questionnaire. We defined children who never had a dog at 6 months, 1.5, and 3 years of age as “never” dog owners; those who had a dog at any of these three time points were defined as “ever” dog owners. Furthermore, “ever” dog owners were categorized into the following three groups: “past only” owners were those who had a dog at both 6 months and 1.5 years of age or either at 6 months or 1.5 years of age but not at 3 years of age; “always” owners had a dog both 6 months and 1.5 years of age or either at 6 months or 1.5 years of age but not at 3 years of age; and “current only” owners did not have a dog at 6 months and 1.5 years of age but did at 3 years of age (Figure 1). 

### 2.6. Outcome Definitions

The ASQ-3 comprises 21 age-specific questionnaires for children aged 1–66 months to assess children’s progress in five developmental domains (communication, gross motor, fine motor, problem-solving, and personal-social). Each of the five domains has six questions, resulting in 30 items for each age interval. Each item describes a skill, ability, or behavior to which the parent responds “yes” (10 points), “sometimes” (5), or “not yet” (0). Parents occasionally omit items when they are unsure of how to respond or because they have concerns about their child’s performance. In this study, the ASQ-3 scores were not calculated if there were three or more omitted items in each domain. In the case of one or two omitted items, an adjusted total domain score was calculated; if a domain had one or two missing items, the domain score was calculated by summing the scores of the remaining items and multiplying it by 1.2 or 1.5, respectively, according to the ASQ-3 manual [16]. Children who may potentially be at risk of developmental delays at each age interval were identified by comparing their scores to cutoff scores. The cutoff scores of the Japanese version of ASQ-3 (J-ASQ-3) for each domain at 3 years of age were as follows: communication: 29.95, gross motor: 39.26, fine motor: 27.91, problem-solving: 30.03, personal-social: 29.89; these cutoff scores were based on previously validated cutoff scores for Japanese children [17]. In this study, children who completed the J-ASQ-3 36 months questionnaire at 34 months and 16 days through 38 months and 30 days of age were strictly included. According to the recommended ASQ-3 procedures, adjusted age was used to determine the appropriate ASQ-3 for children who were preterm (gestational age <37 weeks). 

### 2.7. Statistical Analyses

The ASQ-3 scores of each domain were dichotomized based on their cutoff scores. The chi-square test was used to determine whether there was a significant difference in the frequency of pass/fail of ASQ-3 scores of each domain among the four groups (never, past only, always, and current only). The Kruskal–Wallis test was used to compare the mean maternal age, SF-8 scores (physical component score [PCS] and mental component score [MCS]) [18], birth weight and length, gestational age, and weight and height at 3 years of age among the four groups. Binominal logistic regression models were used to investigate infant developmental delays in association with “never” and “ever” family dog ownerships and then in association with “always” family dog ownership. The models were adjusted for parity, maternal age at delivery, maternal smoking during pregnancy, maternal and paternal education, maternal mental health (MCS), annual family income at ASQ-3 completion, child sex, ETS exposure at 3 years of age, and daycare attendance at 3 years of age, based on previous literature and the correlation between these variables and exposure and outcome. To handle missing covariate values, multiple imputations were applied using the fully conditional specification method. Five imputed datasets were generated based on the assumption that data were missing at random as it has traditionally been suggested to be sufficient on theoretical grounds [19], and pooled exponential parameter estimates were calculated. The following variables were included in the imputation model: parity, maternal age at delivery, maternal smoking during pregnancy, maternal education, paternal education, maternal mental health (SF-8 MCS), family income at ASQ-3 completion, child sex, ETS exposure at 3 years of age, and daycare attendance at 3 years of age. *p* < 0.05 was considered statistically significant. Statistical analyses were performed using SPSS version 26 (IBM, Armonk, NY, USA).

## 3. Results

Table 1 shows the characteristics of the participants sorted by family dog ownership category (*n* = 78,941). Among all the included participants, 83.6% (*n* = 65,986) never owned dogs, 8.5% (*n* = 6745) owned dogs only in the past, 7.0% always owned dogs (*n* = 5556), and 0.9% owned dogs at the current only (*n* = 654). Comparison of parental characteristics found significant differences among the four groups in maternal age, parity, maternal smoking during pregnancy, maternal and paternal education, family income during pregnancy, maternal HRQOL, maternal and paternal smoking, and family income at ASQ-3 completion. Comparison of child characteristics found significant differences among the four groups in gestational age, delivery mode, ETS exposure at 3 years of age, and daycare attendance at 3 years of age. The analysis of participants’ characteristics showed that some of the demographic characteristics of parents and children were different among family dog ownership categories.

Table 2 shows the distribution of ASQ-3 scores in association with the family dog ownership category. The mean scores were 53.06, 55.35, 49.08, 51.75, and 50.33 for the communication, gross motor, fine motor, problem-solving, and personal-social domains, respectively. In total, 3279 (4.2%), 3725 (4.7%), 6186 (7.8%), 5936 (7.5%), and 2735 (3.5%) infants failed the communication, gross motor, fine motor, problem-solving, and personal-social domains, respectively. Comparison of the proportion and pass/fail between “never” and “ever” family dog ownership found that “never” family dog ownership group showed a higher prevalence of fail compared to “ever” family dog ownership in the domains of communication, gross motor, problem-solving, and personal-social. 

Table 3 shows child development delays at 3 years of age in association with “never” and “ever” family dog ownership. The odds ratios of developmental delay in the five domains of ASQ-3 in the “ever” family dog ownership category were compared to that of the “never” family dog ownership. The results found a significantly decreased risk of developmental delay in all domains, except fine motor (odds ratio [OR] = 0.87; 95% confidence interval [CI]: 0.78, 0.96 for communication, OR = 0.83; 95% CI: 0.76, 0.92 for gross motor, OR = 0.89; 95% CI: 0.83, 0.96 for problem-solving, and OR = 0.86; 95% CI: 0.77, 0.97 for personal-social). The result of the complete case analysis of child development delays at 3 years of age in association with “never” and “ever” dog ownership is shown in Table S1.

Table 4 shows child developmental delays at 3 years of age in association with family dog ownership. The odds ratios of developmental delay in the five domains of ASQ-3 in the “past only”, “current only”, and “always” family dog ownership category were compared to that of the “never” family dog ownership. The comparison results showed a decreased odds ratios of developmental delay in the gross motor domain among “past only” (OR = 0.83; 95% CI: 0.73, 0.95) and “always” (OR = 0.86; 95% CI: 0.75, 0.98). In addition, the comparison found that “past only” family dog ownership was associated with a decreased odds ratio of developmental delay in the problem-solving domain (OR = 0.87; 95% CI: 0.79, 0.97), and “always” family dog ownership was associated with a decreased odds ratio of developmental delay in personal-social domain (OR = 0.81; 95% CI: 0.68, 0.95). The result of the complete case analysis of child development delays at 3 years of age in association with family dog ownership is shown in Table S2.

## 4. Discussion

In this study, the association between family dog ownership and early child development was investigated as a primary objective and then various family dog ownership status was further investigated in association with early child development. In this study, we observed that “ever” dog owners showed decreased risks of child development delay in the communication, gross motor, problem-solving, and personal-social domains compared to “never” dog owners. This finding was consistent with our previous findings, which showed that child development at 1 year of age was positively associated with family dog ownership at 6 months of age [12]. All domains, except fine motor, reached statistical significance; “ever” dog ownership showed a reduction of 11%–17% risks compared to “never” dog ownership. The interpretation of the study findings should be done with caution as the magnitude of effect of family dog ownership on child development is very small (OR < 1.68, [20]). Besides, ASQ-3 was a developmental screening tool that pinpoints developmental progress in children, and a reduction of the developmental risks using ASQ-3 did not provide clinical meaning. Furthermore, we observed that family dog ownership at different time periods was associated with decreased risks of child developmental delay in a domain-specific manner. This is the first study to investigate child development in relation to family dog ownership using longitudinal nationwide prospective data. 

The rate of family dog ownership at 3 years of age was 7.9%, which was slightly lower but comparable to the Japanese data of 2019 (12.55%) [21]. This might be related to the fact that participants in the JECS showed a higher prevalence of allergic disease than the general Japanese population, as mentioned in a previous study [22]. The socio-demographic characteristics of “ever” dog owners were different from those of “never” dog owners, particularly in terms of parental characteristics. For example, maternal age at delivery was younger, nulliparous rate was higher, maternal smoking rate during pregnancy was higher, both maternal and paternal education levels were lower, family income during pregnancy and at 3 years of age were lower, and both maternal and paternal smoking rates were higher at 3 years of age in “ever” dog owners than in “never” dog owners (Table 1). Differences in child characteristics were also observed between “ever” and “never” dog owners. ETS exposure and daycare attendance rates were higher among “ever” dog owners than among “never” dog owners. A similar trend in these characteristics was reported in previous studies (Table 1). The findings of this study are consistent with those of previous studies [12,23,24,25]. The association between companion animals, including dogs, and child development has been well discussed in a systematic review [26]. Several potential pathways through which pets may facilitate child development have been suggested. For example, pet attachment can improve a child’s social-cognitive and social-emotional development, and psychosocial health [27,28,29]. Positive attitudes and affiliative interactions with pets may enhance well-being [30]. Owning pets may provide children with opportunities to control their emotions [23].

Child development experts believe that motor activity during the second year is vital to the child’s competence development [31]. This may explain our findings that the gross motor domain was significantly associated with “ever” family dog ownership. In addition, a significantly decreased risk of development delay in the gross motor domain was observed in “past only” and “always” dog owners but not in “current only” dog owners. This study suggests that living with a dog during the critical time period of gross motor development in children may be key to reducing the risk of developmental delay. 

In general, basic fine motor skills gradually develop and are typically mastered between the ages of 6 and 12 in children. In other words, early infancy is not necessarily a crucial period for developing fine motor skills. This is in agreement with our observation of a null association between family dog ownership at early infancy and fine motor development.

To our knowledge, no previous study has investigated child cognitive outcomes in association with family dog ownership in a prospective setting. Cognitive skills encompass problem-solving ability, which was associated with “past” family dog ownership in this study. A systematic review hypothesized that pet attachment enhances social interaction and communication with pets, resulting in a positive influence on social cognition development, and that pet ownership reduces stress levels and improves cognitive executive functions [26]. This was supported by a previous finding of better social-cognitive development in relation to stronger pet attachment [27]. The highest attachment score was reported among dog owners than other pet owners, and children who had a pet dog scored significantly higher on attachment than those who did not [32]. Attachment may be a link between family dog ownership and a decreased risk of developmental delay in the problem-solving domain. According to Bowlby, dogs exhibit behaviors indicative of an attachment relationship [33]. Attachment to the primary caregiver was previously considered to develop over the course of the first 18 months, although the attachment theory later suggested that the critical period for developing an attachment is about 0–5 years [34]. Dog ownership, but not “always” or “current only” family dog ownership, showed a significant association with a decreased risk of developmental delay in the problem-solving domain. Ages between 6 months and 1.5 year were possibly a crucial period for establishing attachment with pet dogs, and the established attachment may have resulted in the decreased risk of developmental delay in children. However, the mechanism for the observed findings is largely unknown, and further exploration is necessary. According to Piaget’s theory, during the sensorimotor stage (up to 2 years), children undergo a period of dramatic growth and learning [35]. In particular, children interact with their environments and go through an astonishing amount of cognitive growth during this period; thus, introducing children to dogs during such a sensitive period may positively influence cognitive development. Considering that communication skills are related to language development, the non-significant reduced risk of developmental delay in the communication domain among “past only” (6 months and/or 1.5 years) dog owners was reasonable as this period was a sensitive period for cognitive development, including language development.

Given that many studies have shown improved levels of mental, emotional, and physical health in dog owners, the influence of dogs on parents should be discussed. One study highlighted the potential of pet dogs to reduce stress in primary carers, including parents of children with an ASD [36]. Other study showed that animal-assisted interventions may provide certain benefits for parents and families during the initial stages of pediatric cancer treatment [37]. Even though these previous studies were limited to the parents of children with medical conditions, the influence of a family dog on parents may improve mental, affective, and other parenting skills, resulting in better child care and better child developmental outcomes.

A relatively large number of studies on socio-emotional development in relation to family dog ownership have been published. A recent longitudinal study found that children with a dog were 20% less likely to have abnormal scores on social-emotional development scales, including emotional symptoms, peer problems, and prosocial behavior, compared to those with no pets [28]. Interacting positively with a dog has been suggested to increase confidence and decrease the fear of rejection in social interactions with other children [38]. Pets can facilitate social interaction between children [4]. However, a prospective study has suggested that a strong bond with a dog results in less time spent with others [39]. Other studies have shown no evidence of an impact of family dog ownership on social developmental outcomes among children aged 4–10 years [29], and on social functioning and psychosocial health of adolescents [40]. The significantly reduced risk of developmental delay in the personal-social domain was found only among “always” dog owners in the current study; this indicates that the duration of time lived with a dog may be important. Together with the previous findings of null impact at older ages, family dog ownership at an early age (<3 years) may be crucial for the socio-emotional development of children. A review has suggested that pet ownership has the greatest influence on children aged <6 years [26]. One potential mechanism by which dogs may confer emotional health benefits to children is the social buffering of stress responses [41]. 

There was no statistically significant association between the risk of development delay in any of the ASQ-3 domains and “current” dog ownership. It should be noted that the “current” group included children who became family dog owners between the age of 1.5 years and 3 years, and the duration of living with a dog ranged from approximately 0 to 18 months; this may hinder any existed association. In addition, the smaller number of participants in the “current” group than in the “past only” and “always” groups may be a reason for wider 95% CIs and null statistical significance. 

This study has some limitations. First, this study assessed family dog ownership status at several points within a very small age range. There appears to be no long-term behavioral benefit from acquiring a pet dog, as child behavior only improved when the child first acquired the dog [29,39]. Studies with more time points and a wider age range are needed to elucidate the impact of the timing of pet acquisition and duration of the time spent with dogs. Second, although we adjusted for possible socio-demographic factors related to family dog ownership status, we did not examine child attachment to dogs. Finally, what was discussed in this study was not based on the direct observation of the child’s relationship with the dog, or their interaction, but only based on the association between family dog ownership and child development. It may be necessary to measure direct relationship and interaction between children and their dogs in a future study to consolidate what was found in this study. 

The strength of the current study is that we used nationwide prospective cohort study data, which represented the general population of Japan. A comparison of the current study population with all JECS participants found that the baseline characteristics, including maternal age at delivery, parity, child sex, birth weight, birth length, gestational age, and delivery mode, were comparable [13]. The proportion of participants with maternal and paternal education ≥13 years was slightly higher among the current study population than in the general Japanese population (63.7% vs. 65.7% for maternal education and 55.8% vs. 57.3% for paternal education), while family income during pregnancy <4 million JPY was slightly lower in the current study population than in the general Japanese population (59.7% vs. 56.8%). Overall, the current study population seemed to be representative of the whole cohort population, and therefore, the selection bias due to the eligibility criteria was considered null or minimal. The longitudinal study design provided high-quality data. However, the findings of this study should be carefully extrapolated because they may not apply to a less generalized population and a population with different cultural backgrounds. The other strength is that we were able to control for multiple socio-demographic factors and maternal HRQOL, which may have an influence on child development. Many of the previous studies showed methodological issues, such as small and non-representative samples and lack of adjustment for confounders; however, the current study could minimize the effects of these issues. Finally, for future investigation, considering the duration of dog ownership may provide further an understanding of the relationship between living with dogs and child development.

## 5. Conclusions

This study showed that living with dogs during early infancy may decrease the risk of developmental delay in the communication, gross motor, problem-solving, and personal-social domains. In addition, the associations were family dog ownership timing specific and were only found in “past only” and “always” groups. Given the possible positive association between early family dog ownership and child development, living with dogs may be an important mechanism to be considered when assessing child development. The current study highlights the need for more longitudinal studies with further follow-ups to examine the possible causal association between family dog ownership and child development.

## Figures and Tables

**Table 1:** Characteristics of participants sorted by family dog ownership category.

			All (*n* = 78,941)	Never (*n* = 65,986)	Ever (*n* = 12,955)			*p*-Value
					Past Only (*n* = 6745)	Current Only (*n* = 654)	Always (*n* = 5556)	
Parental								
Maternal age at delivery (years)			31.5 ± 4.9	31.7 ± 4.9	30.2 ± 5.2	30.0 ± 5.2	31.2 ± 5.2	<0.001
Parity	Nulliparous		32,461 (41.1)	26,458 (40.1)	3264 (48.4)	214 (32.7)	2525 (45.4)	<0.001
	Multipara		44,565 (56.5)	37,982 (57.6)	3293 (48.8)	419 (64.1)	2871 (51.7)	
	Missing		1915 (2.4)	1546 (2.3)	188 (2.8)	21 (3.2)	160 (2.9)	
Maternal smoking during pregnancy	Yes		2699 (3.4)	2013 (3.1)	297 (4.4)	39 (6.0)	350 (6.3)	<0.001
	No		75,275 (95.4)	63,203 (95.8)	6351 (94.1)	603 (92.2)	5118 (92.0)	
	Missing		967 (1.2)	770 (1.1)	97 (1.4)	12 (1.8)	88 (1.6)	
Maternal alcohol consumption during pregnancy	Yes		2092 (2.7)	1717 (2.6)	187 (2.8)	26 (4.0)	162 (2.9)	0.062
	No		75,858 (96.1)	63,464 (96.2)	6473 (96.0)	162 (24.8)	5308 (95.5)	
	Missing		991 (1.3)	805 (1.2)	85 (1.3)	15 (2.3)	86 (1.5)	
Maternal education (years)	<13		26,297 (33.3)	21,062 (31.9)	2671 (40.0)	291 (44.5)	2273 (40.9)	<0.001
	≥13		51,845 (65.7)	44,296 (67.1)	3993 (59.2)	351 (53.7)	3205 (57.7)	
	Missing		799 (1.0)	628 (1.0)	81 (1.2)	12 (1.8)	78 (1.4)	
Paternal education (years)	<13		32,479 (41.1)	26,067 (39.5)	3267 (48.4)	364 (55.7)	2781 (50.1)	<0.001
	≥13		45,272 (57.3)	38,999 (59.1)	3348 (49.6)	274 (41.8)	2651 (47.7)	
	Missing		1190 (1.5)	920 (1.4)	130 (1.9)	16 (2.4)	124 (2.2)	
Family income during pregnancy (million JPY)	<4		28,398 (36.0)	23,514 (35.6)	2527 (37.5)	297 (45.4)	2060 (37.1)	<0.001
	≥4		44,854 (56.8)	38,116 (57.8)	3444 (51.1)	297 (45.4)	2997 (53.9)	
	Missing		5689 (7.2)	4356 (6.6)	774 (11.4)	60 (9.2)	499 (9.0)	
Maternal HRQOL	PCS	Low (<50)	31,166 (39.5)	25,958 (39.3)	2761 (40.9)	240 (36.7)	2207 (39.7)	0.018
		High (≥50)	40,495 (51.3)	34,015 (51.5)	3361 (49.8)	343 (52.4)	2776 (50.0)	
		Missing	7280 (9.2)	6013 (9.1)	623 (9.2)	71 (10.9)	573 (10.3)	
	MCS	Low (<50)	40,367 (51.1)	33,691 (51.1)	3570 (52.9)	348 (53.2)	2758 (49.6)	0.001
		High (≥50)	31,876 (40.4)	26,778 (40.6)	2582 (38.3)	245 (37.5)	2271 (40.9)	
		Missing	6698 (8.5)	5517 (8.3)	593 (8.8)	61 (9.3)	527 (9.5)	
Maternal smoking at 3 years of age	Yes		7312 (9.3)	5557 (8.4)	837 (12.4)	112 (17.1)	806 (14.5)	<0.001
	No		70,953 (89.9)	59,895 (90.8)	5835 (86.5)	533 (81.5)	4687 (84.4)	
	Missing		676 (0.8)	534 (0.8)	73 (1.1)	9 (1.4)	63 (1.1)	
Paternal smoking at 3 years of age	Yes		27,735 (35.1)	22,224 (33.7)	2846 (42.2)	298 (45.6)	2367 (42.6)	<0.001
	No		49,055 (62.1)	42,077 (63.8)	3654 (54.0)	328 (50.2)	2996 (53.9)	
	Missing		2151 (2.7)	1685 (2.6)	245 (3.6)	28 (4.3)	193 (3.5)	
Family income at 3 years of age (million JPY)	<4		24,796 (31.4)	20,148 (30.5)	2505 (37.1)	257 (39.3)	1886 (33.9)	<0.001
	≥4		50,118 (63.5)	42,604 (64.6)	3789 (56.2)	363 (55.5)	3362 (60.5)	
	Missing		4027 (5.1)	3234 (4.9)	451 (6.7)	34 (5.2)	308 (5.5)	
Child								
Sex	Male		40,465 (51.3)	33,845 (51.3)	3443 (51.0)	314 (48.0)	2863 (51.5)	0.380
	Female		38,476 (48.7)	32,141 (48.7)	3302 (49.0)	340 (52.0)	2696 (48.5)	
Birth weight (g)			3011 ± 427	3011 ± 427	3016 ± 421	3019 ± 393	3006 ± 435	0.700
Birth length (cm)			48.9 ± 2.3	48.9 ± 2.3	48.9 ± 2.3	48.9 ± 2.2	48.8 ± 2.4	0.175
Gestational age (weeks)			39.2 ± 1.6	39.2 ± 1.6	39.3 ± 1.6	39.2 ± 1.5	39.2 ± 1.6	0.005
Delivery mode	Vaginal		63,117 (80.0)	52,846 (80.1)	5410 (80.2)	538 (82.3)	4323 (77.8)	<0.001
	Cesarean		15,480 (19.6)	12,849 (19.5)	1309 (19.4)	111 (17.0)	141 (25.4)	
	Missing		344 (0.4)	291 (0.4)	26 (0.4)	5 (0.8)	22 (0.4)	
Weight at 3 years of age (kg)			13.5 ± 2.4	13.5 ± 2.4	13.5 ± 2.1	13.4 ± 1.5	13.5 ± 2.2	0.463
Height at 3 years of age (cm)			91.7 ± 12.9	91.6 ± 12.2	91.7 ± 11.6	92.8 ± 35.2	91.8 ± 17.1	0.066
ETS exposure at 3 years of age	Never/seldom		63,182 (80.0)	53,606 (81.2)	5016 (74.4)	451 (69.0)	4109 (74.0)	<0.001
	Sometimes		12,625 (16.0)	9985 (15.1)	1359 (20.1)	155 (23.7)	1126 (20.3)	
	Often		2432 (3.1)	1818 (2.8)	308 (4.6)	42 (6.4)	264 (4.8)	
	Missing		702 (0.9)	577 (0.9)	62 (0.9)	6 (0.9)	57 (1.0)	
Daycare attendance at 3 years of age	Yes		47,962 (60.8)	39,834 (60.4)	4293 (63.6)	422 (64.5)	3413 (61.4)	<0.001
	No		28,621 (36.3)	24,180 (36.6)	2243 (33.3)	209 (32.0)	1989 (35.8)	
	Missing		2358 (3.0)	1972 (3.0)	209 (3.1)	23 (3.5)	154 (2.8)	

ETS: Environmental tobacco smoke, HRQOL: health-related quality of life, PCS: physical component score, MCS: mental component score. Mean ± standard deviation (SD) or *n* (%). The chi-square test or the Kruskal–Wallis test.

**Table 2:** Distribution of ASQ-3 scores in association with family dog ownership category.

ASQ-3 Domain	Cutoff		All (*n* = 78,941)	Never (*n* = 65,986)	Ever (*n* = 12,955)	*p*-Value
Communication	29.95	Mean ± SD	53.06 ± 55.35	53.03 ± 10.80	53.06 ± 10.73	
		Pass	75,456 (95.6)	63,019 (95.5)	12,437 (96.0)	0.005
		Fail	3279 (4.2)	2800 (4.2)	478 (3.7)	
		Missing	206 (0.3)	167 (0.3)	49 (0.3)	
Gross motor	39.26	Mean ± SD	55.35 ± 8.55	55.29 ± 8.63	55.35 ± 8.55	
		Pass	75,119 (95.2)	62,705 (95.0)	12,414 (95.8)	<0.001
		Fail	3725 (4.7)	3209 (4.9)	516 (4.0)	
		Missing	97 (0.1)	72 (0.1)	25 (0.2)	
Fine motor	27.91	Mean ± SD	49.08 ± 12.86	49.10 ± 12.86	49.08 ± 12.86	
		Pass	72,357 (91.7)	60,504 (91.7)	11,853 (91.5)	0.539
		Fail	6186 (7.8)	5154 (7.8)	1032 (8.0)	
		Missing	398 (0.5)	328 (0.5)	70 (0.5)	
Problem-solving	30.03	Mean ± SD	51.75 ± 10.98	51.71 ± 11.04	51.75 ± 10.98	
		Pass	72,179 (91.4)	60,282 (91.4)	11,897 (91.8)	0.005
		Fail	5936 (7.5)	5040 (7.6)	896 (6.9)	
		Missing	826 (1.0)	664 (1.0)	162 (1.3)	
Personal-social	29.89	Mean ± SD	50.33 ± 10.22	50.28 ± 10.26	50.33 ± 10.22	
		Pass	75,937 (96.2)	63,431 (96.1)	12,506 (96.5)	0.006
		Fail	2735 (3.5)	2339 (3.5)	396 (3.1)	
		Missing	269 (0.3)	216 (0.3)	53 (0.4)	

ASQ-3: Ages and Stages third edition. Mean ± standard deviation (SD) or *n* (%). The chi-square test.

**Table 3:** Child development delays at 3 years of age in association with “never” and “ever” family dog ownership.

	OR (95% CI)	
ASQ-3 Domain	Never	Ever
Communication	1.00	0.87 (0.78, 0.96) **
Gross motor	1.00	0.83 (0.76, 0.92) **
Fine motor	1.00	0.98 (0.91, 1.05)
Problem-solving	1.00	0.89 (0.83, 0.96) **
Personal-social	1.00	0.86 (0.77, 0.97) *

ASQ-3: Ages and Stages third edition, OR: odds ratio, CI: confidence interval. Adjusted for parity, maternal age at delivery, maternal smoking during pregnancy, maternal education, paternal education, maternal mental health (SF-8 MCS), family income at ASQ-3 completion, child sex, environmental tobacco exposure at 3 years of age, and daycare attendance at 3 years of age. * *p* < 0.050, ** *p* < 0.010.

**Table 4:** Child development delays at 3 years of age in association with family dog ownership.

		OR (95% CI)		
ASQ-3 Domain	Never (*n* = 65,986)	Past Only (*n* = 6745)	Current Only (*n* = 654)	Always (*n* = 5556)
Communication	1.00	0.89 (0.78, 1.02)	0.64 (0.40, 1.05)	0.87 (0.75, 1.00)
Gross motor	1.00	0.83 (0.73, 0.95) *	0.66 (0.42, 1.05)	0.86 (0.75, 0.98) *
Fine motor	1.00	0.95 (0.86, 1.05)	0.87 (0.64, 1.20)	1.02 (0.92, 1.13)
Problem-solving	1.00	0.87 (0.79, 0.97) *	0.89 (0.65, 1.22)	0.91 (0.82, 1.02)
Personal-social	1.00	0.92 (0.79, 1.06)	0.83 (0.51, 1.35)	0.81 (0.68, 0.95) *

ASQ-3: Ages and Stages third edition, OR: odds ratio, CI: confidence interval. Adjusted for parity, maternal age at delivery, maternal smoking during pregnancy, maternal education, paternal education, maternal mental health (SF-8 MCS), family income at ASQ-3 completion, child sex, ETS exposure at 3 years of age, and daycare attendance at 3 years of age. * *p* < 0.050.

## Data Availability

Data sharing is not applicable to this article.

## Supplementary Materials

The following are available online at https://www.mdpi.com/article/10.3390/ijerph18137082/s1, Supplemental Table S1 Complete case analysis of child development delays at 3 years of age in association with “never” and “ever” dog ownership. Supplemental Table S2 Complete case analysis of child development delays at 3 years of age in association with family dog ownership.

## Appendix A

Members of the Japan Environment and Children’s Study (JECS) group as of 2020: Michihiro Kamijima (Principal Investigator, Nagoya City University, Nagoya, Japan), Shin Yamazaki (National Institute for Environmental Studies, Tsukuba, Japan), Yukihiro Ohya (National Center for Child Health and Development, Tokyo, Japan), Reiko Kishi (Hokkaido University, Sapporo, Japan), Nobuo Yaegashi (Tohoku University, Sendai, Japan), Koichi Hashimoto (Fukushima Medical University, Fukushima, Japan), Chisato Mori (Chiba University, Chiba, Japan), Shuichi Ito (Yokohama City University, Yokohama, Japan), Zentaro Yamagata (University of Yamanashi, Chuo, Japan), Hidekuni Inadera (University of Toyama, Toyama, Japan), Takeo Nakayama (Kyoto University, Kyoto, Japan), Hiroyasu Iso (Osaka University, Suita, Japan), Masayuki Shima (Hyogo College of Medicine, Nishinomiya, Japan), Youichi Kurozawa (Tottori University, Yonago, Japan), Narufumi Suganuma (Kochi University, Nankoku, Japan), Koichi Kusuhara (University of Occupational and Environmental Health, Kitakyushu, Japan), and Takahiko Katoh (Kumamoto University, Kumamoto, Japan).
