# Supplementary material for: Association between Early Life Child Development and Family Dog Ownership: A Prospective Birth Cohort Study of the Japan Environment and Children’s Study
Source: Int J Environ Res Public Health. 2021 Jul 2;18(13):7082. doi: 10.3390/ijerph18137082 (PMC8295854; doi:10.3390/ijerph18137082)
Supplement: Supplementary file 1 [file ijerph-18-07082-s001.zip › ijerph-1243984-supplementary.pdf]

Table S1. Complete case analysis of child development delays at 3 years of age in association with "never" and "ever" dog ownership.

		OR (95% CI)	
**ASQ domain**	***n***	**Never**	**Ever**
Communication	49,891	1.00	0.89 (0.78, 1.01)
Gross motor	49,950	1.00	0.85 (0.75, 0.96) *
Fine motor	49,813	1.00	0.98 (0.89, 1.07)
Problem-solving	49,598	1.00	0.89 (0.80, 0.98) *
Personal-social	49,872	1.00	0.84 (0.72, 0.97) *

ASQ-3: Age Stages Questionnaire third edition. Never: Never family dog owner, Ever: ever family dog owner. Adjusted for parity, maternal age at delivery, maternal smoking during pregnancy, maternal education, paternal education, maternal mental health (Short Form-8 mental component score), family income at ASQ-3 completed, child sex, environmental tobacco smoke exposure at 3 years-old, daycare attendance at 3 years-old. * $p < 0.050$, ** $p < 0.010$.

Table S2. Complete case analysis of child development delays at 3 years of age in association with family dog ownership.

		OR (95% CI)			
**ASQ domain**	***n***	**Never (*n* = 65,986)**	**Past only (*n* = 6745)**	**Current only (*n* = 654)**	**Always (*n* = 5556)**
Communication	49,891	1.00	0.91 (0.76, 1.08)	0.74 (0.41, 1.32)	0.89 (0.74, 1.06)
Gross motor	49,950	1.00	0.82 (0.69, 0.97) *	0.62 (0.34, 1.13)	0.91 (0.77, 1.08)
Fine motor	49,813	1.00	1.00 (0.88, 1.13)	0.96 (0.65, 1.41)	0.95 (0.83, 1.09)
Problem-solving	49,598	1.00	0.87 (0.76, 0.99) *	0.97 (0.66, 1.43)	0.90 (0.78, 1.03)
Personal-social	49,872	1.00	0.93 (0.77, 1.13)	0.78 (0.41, 1.48)	0.74 (0.59, 0.92) **

ASQ-3: Age Stages Questionnaire third edition. Never: Never family dog owner, Ever: ever family dog owner. Adjusted for parity, maternal age at delivery, maternal smoking during pregnancy, maternal education, paternal education, maternal mental health (Short Form-8 mental component score), family income at ASQ-3 completed, child sex, environmental tobacco smoke exposure at 3 years-old, daycare attendance at 3 years-old. * $p < 0.050$, ** $p < 0.010$.
